# Editorial: The Relationship Between Cognitive Biases and Psychosis: Searching for Mechanisms

**DOI:** 10.3389/fpsyt.2021.753317

**Published:** 2021-09-10

**Authors:** Łukasz Gawęda, Steffen Moritz, Susana Ochoa, Suzanne Ho-wai So

**Affiliations:** ^1^Experimental Psychopathology Lab, Institute of Psychology, Polish Academy of Sciences, Warsaw, Poland; ^2^Department of Psychiatry and Psychotherapy, University Medical Center Hamburg-Eppendorf, Hamburg, Germany; ^3^Parc Sanitari Sant Joan de Déu, Barcelona, Spain; ^4^Mental Health Networking Biomedical Research Center, Centro de Investigación Biomédica en Red de Salud Mental, Madrid, Spain; ^5^The Chinese University of Hong Kong, Hong Kong, SAR China

**Keywords:** psychosis, cognition, cognitive bias, mechanisms, risk of psychosis

The development of cognitive psychology and the influence of its principles on other branches of psychology has revolutionized our understanding and treatment of psychiatric disorders. From the very beginning, the cognitive perspective on psychopathology was in opposition to the psychoanalytical model by offering scientifically falsifiable models and shortly after its introduction clinical interventions. Cognitive models of psychopathology have evolved in different directions and have been successfully adapted to very different psychiatric disorders, suggesting that it may be a transdiagnostic approach (1). Despite the variations in specific cognitive models of mental disorders, dysfunctional information processing, i.e. cognitive biases, are placed in the central focus in this approach. Biased information processing has been implicated in the development and maintenance of psychopathological symptoms across very different psychiatric disorders. What is even more important, studies have successfully demonstrated that the cognitive approach is clinically useful showing that cognitive interventions ameliorate cognitive biases and that these changes often transfer to symptom reduction (2, 3).

Unfortunately, psychosis and more notably schizophrenia spectrum psychoses have long been viewed as being beyond psychological understanding and treatment. It should be noted that as early as 1952 Beck (4) presented successful cognitive strategies that he used in working with a patient with paranoia around the time that chlorpromazine was introduced as the very first antipsychotic agent. However, the development of new medications and the negation of a psychological understanding of psychotic symptoms have prolonged the development of cognitive approaches to psychosis to the mid-'90s (5).

In the last three decades, we have been witnessing the systematic development of cognitive approaches to psychosis in general or its different specific symptoms like delusions, hallucinations, and negative symptoms. Again, all these models have placed biased information in the very center of their interest. In the late ‘80s, Frith and Done (6) followed later by Bentall (7) have proposed that dysfunctions in distinguishing between internal and external sources of information may constitute a cognitive background for the development and maintenance of some of the positive symptoms of schizophrenia (e.g., auditory hallucinations). Other researchers have focused their attention on biased tendencies in information processing that may give rise to delusional thinking (8). First studies in the late'80s and early'90s have provided the first empirical evidence that patients with schizophrenia exhibit cognitive biases to a much larger extent than healthy controls. Both evidence for source monitoring problems [e.g., (9)] and jumping to conclusions [e.g., (10)] have been confirmed several times in meta-analyses. These studies were the beginning of “madness [being] explained” (11) by psychological models. Recently, there are hundreds of studies on the role of different cognitive biases in psychosis, its risk states, and specific psychotic symptoms. This line of research has stimulated the development of different cognitive-behavioral therapies proven to be effective in psychosis.

Nevertheless, despite a growing number of studies on cognitive biases in psychosis, the mechanisms of how cognitive biases increase the risk of psychosis is still not clear. What is more, how cognitive biases that are important for psychosis arise is also relatively unknown. Hence, within the scope of the recent Research Topic, we intended to gather different studies addressing the mechanisms of the relationships between dysfunctional information processing and psychosis as well as its risk states.

In line with existing knowledge, current studies (this issue) have confirmed that exaggerated cognitive biases are observed along the continuum of psychosis from psychotic-like experiences to full-blown psychosis. The importance of assessment is relevant, in this way one of the manuscripts is centered on the validation of a tool for the assessment of cognitive biases in schizophrenia (Corral et al.). Importantly, studies published in the current Research Topic extended our understanding of the relationships between different cognitive biases and psychosis. Prochwicz et al. explained the mechanisms of the relationship between attention to threat and the non-clinical risk of psychosis, showing that the linkage is mediated by emotional stress. Intriguingly, the authors offered a further explanation by showing that the indirect effect of attention to threat via stress on psychotic-like symptoms was significantly moderated by higher levels of distraction-seeking coping strategies. In another study, Pytlik et al. aimed at a better understanding of the relationship between data gathering strategies and preclinical risk of psychosis. The authors have found that a tendency to jump to conclusions is related to conspiracy theories, which may be conceptualized on a continuum with paranoia as one extreme pole. Interestingly, Pytlik et al. have found that a higher tendency to engage in intuitive thinking, which is followed by data gathering biases, may constitute a risk for stronger belief in conspiracy theories. Data gathering and weighing biases as a cognitive underpinning of psychosis have also been investigated by Scheunemann et al. The authors found that individuals with more frequent psychotic-like experiences tend to not only overestimate their initial decision but also weigh currently available advice given in a decision-making task more strongly. García-Mieres et al. showed that a higher level of cognitive rigidity in an interpersonal context may be a marker of worse functioning for patients across a wide range of different domains (e.g., executive functions, higher self-certainty, earlier age of the onset). Zhu et al. also found that reduced cognitive flexibility is related to the severity of delusions. Interestingly, a tendency for a biased response to contradiction was higher among patients with delusion as compared to patients with depression. Taken together, these studies suggest that the preclinical risk of psychosis may be related to biases in different aspects of data gathering and weighting. Hence, published studies suggest that patients with psychotic symptoms, especially those with delusions, might be less cognitively flexible and may react in a biased way to contradiction, which may be a cognitive factor in maintaining the symptoms (this issue).

In Seo et al. another aspect of cognitive biases, i.e., facial recognition, was investigated among patients fulfilling clinical risk states for psychosis. They found that inaccuracy in emotion recognition may be an early marker for the risk of psychosis. Interestingly, a disgust response to neutral faces was related to the severity of paranoia. Furthermore, in another study investigating social cognition, Dorn et al. found that deficits in cognitive Theory of Mind may be specifically linked to general delusion severity, while emotional Theory of Mind was more closely related to negative and disorganized symptoms. These studies confirmed our prior knowledge on the important role of deficits in social cognition as a risk factor for psychosis (12).

Extending prior studies, Rodriguez's et al. narrative review discussed the interaction between exposure to life adversities, cognition, and social cognition, in contributing to psychosis. This review was based on the recent theoretical account of the risk of psychosis (13) as a dynamic interaction between social genetic predisposition, social adversities, and cognition. An interesting study was presented by Pionke-Ubych et al. in which cognitive biases were examined together with exposure to life-adversities and self-disturbances in a non-clinical sample with frequent psychotic-like experiences. Confirming prior studies [e.g., (14)], they found that indeed early childhood trauma may have an indirect effect on the risk of psychosis via biased information processing as well as self-disturbances. These findings provide an interesting contribution to the field by showing dynamic interactions between environmental factors, cognition, and self-disturbances that have all been shown to increase the risks of developing psychosis.

To sum up, we believe that the papers published in this Research Topic provide an interesting contribution to our understanding of the role of cognitive biases in psychosis and its risk states. Riding on the literature that focused on cognitive biases in schizophrenia over the last three decades, this Research Topic has further explored mechanisms of action and extended the investigation from patient samples through to the risk of psychosis. The presented articles also endeavored to address how different information processing biases may contribute to psychosis in the context of environmental factors, emotional processes, and interactions with other cognitive processes. Yet, our knowledge is still incomplete. The newest studies in the field have suggested the interaction between genetic, environmental factors, and cognitive biases in predicting the risk of psychosis [e.g., (15)]. Hence, further integration is warranted to gather new insights that may apply to clinical practice.

## Author Contributions

ŁG wrote the first version of the editorial. All authors edited and contributed to the final version.

## Conflict of Interest

The authors declare that the research was conducted in the absence of any commercial or financial relationships that could be construed as a potential conflict of interest.

## Publisher's Note

All claims expressed in this article are solely those of the authors and do not necessarily represent those of their affiliated organizations, or those of the publisher, the editors and the reviewers. Any product that may be evaluated in this article, or claim that may be made by its manufacturer, is not guaranteed or endorsed by the publisher.
